# Melioidosis in Travelers Returning from Vietnam to France

**DOI:** 10.3201/eid2209.160169

**Published:** 2016-09

**Authors:** Jérôme Gauthier, Patrick Gérôme, Maryline Defez, Fabienne Neulat-Ripoll, Barbara Foucher, Thierry Vitry, Lionel Crevon, Eric Valade, François M. Thibault, Fabrice V. Biot

**Affiliations:** Hôpital d’Instruction des Armées Desgenettes, Lyon, France (J. Gauthier, P. Gérôme, B. Foucher, T. Vitry, L. Crevon);; Centre Hospitalier Universitaire, Grenoble, France (M. Defez);; Institut de Recherche Biomédicale des Armées, Brétigny-sur-Orge, France (F. Neulat-Ripoll, E. Valade, F.V. Biot);; Ecole du Val-de-Grâce, Paris, France (E. Valade);; Direction Centrale du Service de Santé des Armées, Paris (F.M. Thibault)

**Keywords:** melioidosis, *Burkholderia pseudomallei*, cervical lymphadenitis, MALDI-MS, outbreak, travel medicine, Vietnam, bacteria, bacterial infection, France

**To the Editor:** Melioidosis, a potentially fatal infectious disease, occurs predominantly across much of Asia and in northern Australia because of the soil and water bacterium *Burkholderia pseudomallei* (*1*). We report 2 related cases of suppurative cervical lymphadenitis, an unusual adult presentation of melioidosis, in 2 men who returned to France from Vietnam on the same trip (*2*).

Patient 1, a 28-year-old previously healthy man, was admitted to our hospital in Lyon, France, in October 2013 for the evaluation of a palpable neck mass, which had been growing steadily for the previous 2 months. Examination of the head and neck revealed a fluctuant, tender mass located in the inferior angle of the right side of the mandible, mimicking lymph node tuberculosis. Ultrasonographic investigation confirmed a level II enlarged cervical lymph node that was 2.8 cm in diameter. The routine bacterial culture from an ultrasound-guided fine needle aspiration showed a microorganism that was identified as *B. pseudomallei* by matrix-assisted laser desorption/ionization time-of-flight mass spectrometry analysis with an extended database. An investigation by local medical staff revealed that the symptoms of our patient began in August 2013, when he returned to France after attending a wedding ceremony in Vietnam, a country to which *B. pseudomallei* is known to be endemic (*3*). No environmental risk factors, such as the percutaneous inoculation of contaminated material, ingestion, or inhalation, which are the main routes of transmission of melioidosis, were reported (*1*). The interview of patient 1 identified a co-traveler with similar symptoms (patient 2), who was subsequently admitted to the same hospital. 

Patient 2, a 31-year-old previously healthy man, reported a 2-month history of a painful, inflamed, gradually enlarging, right-sided neck mass, accompanied by weight loss, night sweats, and intermittent fevers. Examination showed an enlarged cervical lymph node that was confirmed as level III, 3 cm in diameter. After noncontributory culture results from an ultrasound-guided fine needle aspiration, we performed an open biopsy under general anesthesia. We excised an adherent, enlarged, pus-filled lymph node and necrotic tissue for microbiologic testing. A real-time PCR assay specifically targeting type-3 secretion system genes (*orf11* and *BpSCU2*) quickly revealed the presence of *B. pseudomallei* DNA, and the diagnosis of melioidosis was confirmed by culture, matrix-assisted laser desorption/ionization time-of-flight mass spectrometry, and antibiogram (*4*). 

For both patients, a cervical, chest, abdominal, and pelvic computed tomographic scan showed no other foci of infection, and a 14-day regimen of intravenous ceftazidime therapy was administered, followed by oral treatment with cotrimoxazole for 6 months (*5*). However, the neck mass of patient 1 was still swollen after 1 month of treatment, and oral amoxicillin/clavulanic acid was added to cotrimoxazole for 2 months based on an antibiogram from a new bacterial isolation. For patient 2, cotrimoxazole was switched to amoxicillin/clavulanic acid after a presumed adverse drug reaction. At last report, both patients had been disease free for 20 months.

As soon as the second case of melioidosis was confirmed, local and national public health authorities in France were notified, and a larger contact investigation was initiated because 16 other travelers attended the same wedding ceremony before returning to their home countries. None of these travelers had any symptoms of melioidosis. No serologic testing was performed. 

Although sporadic cases of travel-associated melioidosis are regularly reported, such case clusters occurring in returning travelers is rare but underscores the role of the contact investigation in this context (*6**–**8*). Phylogenetic analyses, performed by a 7-locus multilocus sequence typing analysis, revealed that the 2 isolates shared the same sequence type (381) (identification nos. 4488 and 4489, http://pubmlst.org/bpseudomallei), which was previously identified in Thailand and Cambodia (*9*), suggesting a clonal infection from a single-point source. The epidemiologic assessment will be completed by whole-genome sequencing. 

Most cases of oropharyngeal melioidosis have been reported in children and were believed to be associated with an oral contamination (*1*,*3*). The ingestion of unchlorinated or inefficiently chlorinated water from local residences and hotels has been involved in melioidosis outbreaks and could have been the route of infection for these patients. However, the source of infection might also be linked to a scooter ride taken by both men together around a lake in the vicinity of Hanoi (*10*). 

These patients had no known individual risk factor for melioidosis, such as diabetes, hazardous alcohol use, chronic lung or renal disease, thalassemia, glucocorticoid and other immunosuppressive therapy, or cancer, whereas up to 70% of patients with travel-associated melioidosis had >1 predisposing factor (*1*,*8*). However, the percentage of patients with an underlying risk factor dropped to 37.5% when the data excluded patients who were born in melioidosis-endemic countries or others who had a long-term stay in a melioidosis-endemic country (*6*). This finding makes us cautious not to repeat making the common assumption about the link between underlying conditions and the risk for melioidosis, especially in regard to conventional tourists traveling in melioidosis-endemic areas.
